# Association between income levels and prevalence of heat- and cold-related illnesses in Korean adults

**DOI:** 10.1186/s12889-021-11227-4

**Published:** 2021-06-29

**Authors:** Jin-young Min, Hyeong-Seong Lee, Yeon-Soo Choi, Kyoung-bok Min

**Affiliations:** 1 Veterans Medical Research Institute, Veterans Health Service Medical Center, Seoul, Republic of Korea; 2 Department of Statistics and Data Science, College of Commerce and Economics, Yonsei University, Seoul, Republic of Korea; 3 Department of Preventive Medicine, College of Medicine, Seoul National University, Seoul, Republic of Korea; 4 Institute of Health Policy and Management, Seoul National University Medical Research Center, Seoul, Republic of Korea

**Keywords:** Heat wave, Cold spell, Climate change, Illnesses, Thermoregulation

## Abstract

**Background:**

Given that low income worsens health outcomes, income differences may affect health disparities in weather-related illnesses. The aim of this study was to investigate the association between income levels and prevalence of heat- and cold-related illnesses among Korean adults.

**Methods:**

The current study comprised 535,186 participants with all variables on income and health behaviors. Patients with temperature-related illnesses were defined as individuals with outpatient medical code of heat- and cold-related illnesses. We categorized individual income into three levels: “low” for the fourth quartile (0–25%), “middle” for the second and the third quartiles (25–75%), and “high” for the first quartile (75–100%). To examine income-related health disparities, Cox proportional hazard regression was performed. Hazard ratios (HRs) and 95% CI (confidence interval) for heat- and cold-related illnesses were provided. The model adjusted for age, sex, smoking status, alcohol drinking, exercise, body mass index, hypertension, hyperglycemia, and local income per capita.

**Results:**

A total of 5066 (0.95%) and 3302 (0.62%) cases identified patients with heat- and cold-related illnesses, respectively. Compared with high income patients, the adjusted HR for heat-related illnesses was significantly increased in the low income (adjusted HR = 1.103; 95% CI: 1.022–1.191). For cold-related illnesses, participants with low income were likely to have 1.217 times greater likelihood than those with high income (95% CI: 1.107–1.338), after adjusting for other covariates. In the stratified analysis of age (20–64 years and over 65 years) and sex, there was no difference in the likelihood of heat-related illnesses according to income levels. On the other hand, an HR for cold-related illnesses was higher in patients aged 20 to 64 years than in those aged over 65 years. Male with low income had also a higher HR for cold-related illnesses than female with low income.

**Conclusions:**

Our results showed that heat- or cold-related illnesses were more prevalent in Koreans with low income than those with high income. Strategies for low-income subgroups were needed to reduce greater damage due to the influence of extreme temperature events and to implement effective adaptation.

## Background

Income is a pervasive issue in public health. Many cross-sectional and longitudinal studies have suggested that poverty is one of the strongest predictors of poor health outcomes [1, 2]. Plausible theories on the direct and indirect effects of income on health have been proposed [1, 3]. Direct effects are observed when individuals’ health is affected only by their own income [3]. The difference of individual income can increase inequality in health-producing material resources (e.g., nutrition and housing conditions), leading to larger health disparities between the rich and the poor [1]. Indirect effects work through changes in other individual’s incomes [3]. Moreover, changes in social, political, economic, and cultural circumstances have been found to alter the relationship between individual income and individual health, even if their income remains unchanged [3]. Eventually, the difference in individual income imposes a health risk by strengthening the direct and indirect causal processes in health disparities rather than being a new independent determinant of health [1–3].

Income-related health disparity is an important consideration under extreme weather and climate change conditions [4, 5]. Although extreme weather and climate events affect individuals worldwide, they may not be distributed equally or fairly [5–7]. For example, those living in low-income communities and countries are more likely to suffer from or die of climate-sensitive diseases (i.e., vector-borne and water-borne illness) relative to their high-income counterparts considering that poverty limits material resources and capacity to cope with adverse climatic conditions [8]. Within developing and developed countries, poor people tend to be more affected by climate-related shocks (i.e., cold and heat, flooding, and droughts) and be at greater risks of adverse health outcomes caused by climate hazards [4, 5, 7]. In the contextual approach, income inequity exacerbates health risks by increasing vulnerability to a given hazard [4].

The term “vulnerability” has been conceptualized in various different ways in different fields. Herein, vulnerability is not simply poverty but is seen as the outcome of a combination of “exposure”, “sensitivity”, and “adaptive capacity” derived from differences in individual income [6, 7]. The high vulnerability of the poor is based on the lack of health-producing material resources (namely, the direct effect of unequal income) and by changes in social, political, economic contexts, and culture (namely, the indirect effect of unequal income) [5, 6]. For example, impoverished people are more likely to live in flood-prone areas, die during heat waves, and have limited abilities to cope from the loss and damage caused by climate change [5, 6]. As such, fortuneless individuals face disproportionately higher risks from extreme weather and climate changes, while affluent individuals are generally placed in less vulnerable, resilient environments [6, 7].

Under climate change scenarios, extreme temperatures - like heat waves and cold spells - have received much attention because of their adverse health impacts, especially heat- and cold-related mortality [8–11]. Researchers have identified various subgroups that have a higher vulnerability to extreme temperatures [11–15]. Several socioeconomic variables (e.g., age, educational attainment, occupation, and marital status) have been considered as important effect modifiers in the impact of extreme temperatures on mortality [11–15]. In an extreme thermal environment, the elderly have been consistently identified to be at higher risk of all-cause and cause-specific mortality, possibly due to their reduced thermal regulatory function, multiple comorbidities, and limited access to healthcare [16, 17]. Individuals who were less educated, had lower employment, and were unmarried exhibited a significant increases in mortality risk following exposure to extremely hot or cold temperatures [11–15].

Most studies have thus far been conducted on mortality attributable to heat or cold [11–15, 17]. Exposure to extreme or prolonged heat or cold can produce several health effects through the failure of thermoregulatory mechanisms, causing heat- and cold-related illnesses, such as heatstroke, heat exhaustion, hypothermia, and frostbite [18]. Given the above-mentioned income inequality and health disparities due to climate hazards, individual income may affect the occurrence of heat- and cold-related illnesses. Nonetheless, little is known regarding the impact of unequal income on heat- or cold-related illnesses. In this study, we hypothesized that low-income individuals are more likely to have illnesses related to heat or cold stress than high-income individuals. We examined the association between income levels and the prevalence of heat- and cold-related illnesses in Korean adults.

## Methods

### Study population

This study used the 2002–2015 National Health Insurance Service-National Sample Cohort (NHIS-NSC). The NHIS-NSC contains medical records (i.e., records of diagnosis, treatments, and surgery), prescription details, and medical expenses provided by healthcare facilities including inpatient, outpatient, and emergency departments. It also contains details of participants’ identity, socioeconomic status (SES), and health examinations [19]. Briefly, the NHIS-NSC is a population-based cohort based on National Health insurance services (NHIS) in South Korea. The NHIS-NSC consisted of a representative sample cohort of 2.2% (about 1 million subjects) of the entire population in 2002 established by a stratified random selection from the NHIS. The cohort lasted for 13 years until 2015, unless a participant was disqualified due to death or immigration. During 2002 to 2015, since the cohort size reduced due to several disqualifications, it was refreshed by adding a representative sample of newborns. According to the Cohort profile article, the NHIS-NSC data was constructed by systematic stratified random sampling with proportional allocation within each stratum. Specifically, strata was constructed by age groups (infants under 1 year, ages 1–4, 5-year age groups between 5 and 79, and 80 years and above), sex (male; female), participant’s income level (upper 20 percentiles for insured employees, lower 20 percentiles for insured self-employed individuals, and the lowest level of income for medical aid beneficiaries). The stratified sample was extracted repeatedly until the maximum absolute percentage error, defined as the relative percentage difference between the population and the sample mean of total annual health care cost to the population mean, reached a predefined value of less than 5%. Although the representativeness for follow-up years is not perfectly guaranteed, the use of a suitable and sufficient sample size for the initial cohort ensured representativeness. Therefore, sample representation was evaluated by examining whether the sample contained a population average in the confidence interval of 95% of the average annual medical expenses [19]. The generation and overview of data is described in detail in previous papers [19].

From the NHIS-NSC (project number: NHIS-2016-2-0081 and NHIS-2019-2-231), we identified 721,630 adults ages ≥20 years in 2002. Of them, 29,013 subjects without information on household income were precluded, and 692,617 subjects were initially recruited. We further excluded 186,444 subjects with no data on health-behavior variables (i.e., smoking status, alcohol drinking, and exercise) and no height or weight measurements. Finally, the current study comprised 535,186 participants. The Institutional Review Board of the Seoul National University Hospital approved the study protocol, and informed consent was exempted by the committee (IRB number: E-1907-053-1046). Informed consent from each patient was waived since the NHIS-NSC was anonymous data. All methods were carried out in accordance with relevant guidelines and regulations.

#### Definition of heat or cold-related illnesses

Outcome variables were obtained from the NHIS-NSC and included heat- or cold-related illnesses. The definition of disease was based on the tenth revision of the International Statistical Classification of Diseases and Related Health Problems (ICD-10). A case of heat-related illness was defined as an individual with outpatient medical code of T67, which describes the diagnosis of the “effects of heat and light.” A cold-related illness was defined as an individual having the diagnostic code of T68.0 (Hypothermia), T69.0 (Immersion hand and foot), T69.1 (Chilblains), T69.8 (Other specified effects of reduced temperature), T69.9 (Effect of reduced temperature, unspecified), T33.0–9 (Superficial frostbite), T34.0–9 (Frostbite with tissue necrosis), and T35.0–7 (Unspecified frostbite).

#### Variables of interest

A key variable was income. The NHIS-NSC provided subjects’ income levels as percentiles instead of original continuous income value. We categorized income levels into three levels: “low” for the fourth quartile (0–25%), “middle” for the second and the third quartiles (25–75%), and “high” for the first quartile (75–100%).

**Fig. 1:**
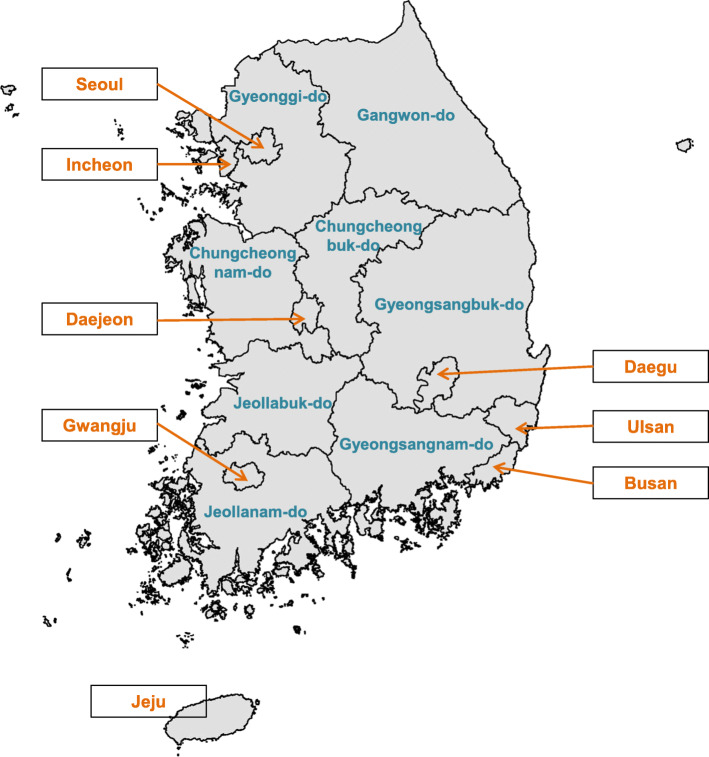
Administrative districts in Republic of Korea

Other variables of interest included subjects’ demographics, health behaviors, and health condition. Demographic variables included age (stratified by 5-year intervals: 20–24, 25–30, …, or 75+) and sex, while health behaviors comprised smoking status (current, past, or never a smoker), alcohol consumption (yes or no), and regular exercise (yes or no). For health condition, the body mass index (BMI) was calculated by a person’s weight in kilograms divided by the square of height in meters. The BMI was categorized into an underweight group (BMI ≤ 18.5 kg/m^2^), normal group (18.5 kg/m < BMI ≤ 25 kg/m^2^), overweight group (25 < BMI ≤30 kg/m^2^), and obese group (BMI over 30 kg/m^2^) [20]. High blood pressure was assigned when their highest blood pressure was greater than 140 mm/hg or lowest blood pressure was greater than 90 mm/hg. Hyperglycemia was designated when their blood sugar was reported over 125 mg/dl. The average local income per capita was included to explain economic gap by administrative districts, which are governmental area units for local administration (Fig. 1).

#### Statistical method

Chi-square test was applied to assess the statistical discrepancy in participants’ characteristics by the presence of heat- and cold-related illnesses. To estimate the risk of developing heat- and cold-related illnesses, ‘person-years’ were calculated. The person-years were accumulated from 2002 until the year of the illness diagnosis or until the end of the study: participants with heat- and cold-related illnesses were followed up until the year of diagnosis, while those not diagnosed with the illnesses were followed up until the end of the study period. To examine income-related health disparities, multiple regressions of heat- and cold-related illnesses on the income level were performed. The Cox proportional hazard model provided the hazard ratio (HR) and its 95% confidence interval (95% CI) of heat- and cold-related illnesses as increases in the income level. The high-income level was set as a reference. The association between income and extreme temperature-related illnesses was adjusted for age (stratified by 5-year intervals), sex, smoking status, alcohol consumption, exercise, BMI categories, high blood pressure, hyperglycemia, and local income per capita. Moreover, because of distributional heterogeneities in age and sex, we conducted stratified analysis by age and sex. All the analyses were performed using SAS 9.4 software (SAS Institute, Cary, NC, USA). Throughout our study, statistical significance was based on the 0.05 threshold significance level.

## Results

We divided patients with heat- or cold-related illnesses according to their characteristics of demographic, health behaviors, and health conditions and cross-tabulated them according to income levels, as shown in Table 1. Of the participants, 5066 (0.95%) and 3302 (0.62%) cases were defined as patients with heat- and cold-related illnesses, respectively. Then we calculated per 10,000 incidence rates of heat- and cold-related illnesses based on corresponding person-years. For heat-related illnesses, the incidence rates were statistically different by age group and gender, regardless of income levels. For cold-related illnesses, there was a significant difference in incidence rates between the male and female groups. This difference was consistently significant across all levels of income. The incidence rate of the overweight group (95% CI: 4.5–5.8) was significantly less than underweight (95% CI: 7.0–11.2), and normal-weight group (95% CI: 7.1–8.1). For a group of age from 20 to 64, people with low income level (95% CI: 6.5–7.4) were at significantly higher risk than those with higher income levels (95% CI: 5.5–6.1 for middle income level, and 4.9–5.8 for high income level). This kind of susceptibility was also detected for males; never smokers; people who exercise; people with normal weight; people who do not have high blood pressure; and people without hyperglycemia. Local income per capita within each administrative division during the study period was calculated. The average local income per capita (10,000 KRW) by administrative districts was as follows: Seoul (15,048.6), Busan (11,985.7), Daegu (11,752.7), Incheon (11,395.7), Gwangju (11,930.1), Daejeon (12,438.5), and Ulsan (15,503.2), Gyeonggi-do (12,824.6), Gangwon-do (11,105.6), Chungcheongbuk-do (11,392.1), Chungcheongnam-do (11,504.5), Jeollabuk-do (11,439.5), Jeollanam-do (11,483.8), Gyeongsangbuk-do (11,702.1), Gyeongsangnam-do (12,190.1), and Jeju Special Self-governing Province (11,731.5).

**Table 1:** Incidence rate (95% CI) of heat- and cold-related illnesses by participants’ characteristics

Income level	10,000 Person-year			Heat-related illnesses			Cold-related illnesses		
	Low	Middle	High	Low (*n* = 1,618)	Middle (*n* = 2,279)	High (*n* = 1,169)	Low (*n* = 1,172)	Middle (*n* = 1,437)	High (*n* = 731)
Age (year)									
20–64	152.4	227.9	121.2	9.1 (8.6–9.5)	8.6 (8.3–9.0)	8.0 (7.5–8.5)	6.9 (6.5–7.4)	5.8 (5.5–6.1)	5.3 (4.9–5.8)
65+	12.8	18.8	14.1	18.6 (16.2–20.9)	16.6 (14.8–18.4)	14.3 (12.3–16.3)	6.0 (4.7–7.3)	5.7 (4.7–6.8)	5.9 (4.6–7.1)
Sex									
Male	75.5	125.1	69.1	8.5 (7.8–9.1)	8.2 (7.7–8.7)	7.3 (6.7–8.0)	6.1 (5.5–6.6)	4.9 (4.5–5.3)	4.5 (4.0–5.0)
Female	89.8	121.6	66.2	10.9 (10.2–11.6)	10.3 (9.7–10.8)	10.0 (9.3–10.8)	7.5 (7.0–8.1)	6.8 (6.3–7.3)	6.3 (5.7–6.9)
Smoking status									
Current smoker	46.5	67.0	30.6	7.7 (6.9–8.5)	8.0 (7.3–8.7)	7.6 (6.6–8.6)	5.3 (4.7–6.0)	4.5 (4.0–5.0)	4.3 (3.5–5.0)
Former smoker	12.7	22.6	14.1	9.8 (8.0–11.5)	7.4 (6.3–8.5)	7.0 (5.6–8.4)	6.9 (5.4–8.3)	5.1 (4.2–6.0)	4.4 (3.3–5.5)
Never smoker	106.1	157.1	90.6	10.7 (10.1–11.4)	10.0 (9.5–10.5)	9.2 (8.6–9.9)	7.5 (7.0–8.1)	6.5 (6.1–6.9)	6.0 (5.4–6.5)
Current alcohol consumption									
No	86.8	128.9	73.5	11.1 (10.4–11.8)	10.5 (10.0–11.1)	9.5 (8.8–10.2)	7.3 (6.8–7.9)	6.2 (5.7–6.6)	5.8 (5.3–6.4)
Yes	78.5	117.8	61.8	8.3 (7.7–8.9)	7.8 (7.3–8.3)	7.6 (6.9–8.3)	6.3 (5.8–6.9)	5.4 (5.0–5.9)	4.9 (4.4–5.5)
Exercise									
No	99.4	140.6	68.3	10.5 (9.8–11.1)	9.8 (9.3–10.3)	9.5 (8.8–10.3)	6.9 (6.3–7.4)	6.2 (5.8–6.6)	6.0 (5.4–6.6)
Yes	65.9	106.1	67.0	8.8 (8.0–9.5)	8.5 (8.0–9.1)	7.7 (7.1–8.4)	6.9 (6.2–7.5)	5.3 (4.9–5.7)	4.8 (4.2–5.3)
BMI categories									
Underweight	8.1	9.7	4.7	9.8 (7.7–12.0)	7.6 (5.9–9.4)	11.6 (8.5–14.7)	9.1 (7.0–11.2)	8.9 (7.0–10.7)	8.1 (5.5–10.7)
Normal weight	106.3	157.2	85.4	9.5 (8.9–10.1)	9.2 (8.7–9.6)	8.4 (7.8–9.0)	7.6 (7.1–8.1)	6.6 (6.2–7.0)	6.0 (5.5–6.6)
Overweight	44.4	70.8	40.7	10.4 (9.4–11.4)	9.6 (8.9–10.4)	8.8 (7.9–9.7)	5.1 (4.5–5.8)	4.1 (3.7–4.6)	4.0 (3.4–4.6)
Obese	6.4	9.1	4.5	10.5 (8.0–13.1)	8.8 (6.9–10.8)	8.8 (6.1–11.6)	3.8 (2.3–5.3)	2.9 (1.8–4.0)	2.9 (1.3–4.4)
High Blood Pressure									
No	143.7	215.2	117.8	9.7 (9.2–10.2)	9.1 (8.7–9.5)	8.5 (7.9–9.0)	7.2 (6.7–7.6)	6.1 (5.7–6.4)	5.6 (5.2–6.1)
Yes	21.5	31.5	17.5	10.4 (9.0–11.7)	10.1 (9.0–11.2)	9.8 (8.3–11.2)	4.9 (4.0–5.9)	4.2 (3.5–4.9)	3.8 (2.9–4.7)
Hyperglycemia									
No	154.8	231.4	126.4	9.7 (9.2–10.2)	9.1 (8.8–9.5)	8.6 (8.1–9.1)	6.9 (6.4–7.3)	5.9 (5.6–6.3)	5.5 (5.1–5.9)
Yes	10.4	15.3	8.9	10.9 (8.9–13.0)	10.6 (8.9–12.2)	9.1 (7.1–11.1)	6.8 (5.2–8.4)	4.0 (3.0–5.0)	4.6 (3.2–6.0)

*BMI* body mass index

For heat-related illnesses, the proportion of the patients increased with age such that a large number of patients aged over 65 years developed the same. Regarding health conditions, a higher proportion of patients with high blood pressure or hyperglycemia were found to acquire heat-related illnesses. Furthermore, patients with cold-related illnesses were more likely to be diagnosed at a younger age compared to those without the illnesses. The proportion of cold-related illnesses was higher among underweight subjects and those without medical conditions such as high blood pressure and hyperglycemia. Similarly, patients with heat- or cold-related illnesses were more likely to be female, have low income, be non-smokers and non-drinkers, and exercise lesser than their counterparts.

**Fig. 2:**
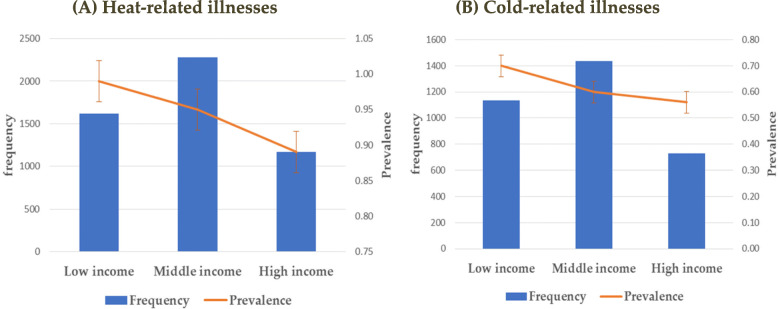
Frequency and prevalence of heat- and cold-related illnesses according to income level: (**A**) Heat-related illnesses and (**B**) Cold-related illnesses

Figure 2 displays the distribution of patients with heat- or cold-related illnesses according to income levels. Overall, patients with these illnesses were disproportionally distributed according to income levels, with low-income patients having being the highest prevalence. The number (prevalence) of patients with heat-related illnesses was 1618 (0.99), 2279 (0.95), and 1169 (0.89) for the low-, middle, and high-income groups, respectively. Regarding cold-related illnesses, the numbers and prevalence were 1134 (0.66), 1437 (0.60), and 731 (0.56) respectively for the low-, middle-, and high-income groups, respectively.

**Fig. 3:**
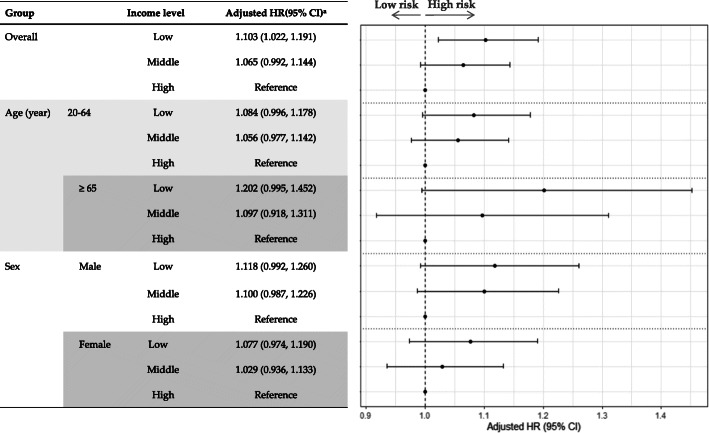
Adjusted hazards for heat-related illnesses according to income levels. ^a^ Adjusted model was controlled for age, sex, smoking status, alcohol drinking, exercise, BMI group, hypertension, hyperglycemia, and local income per capita

Figure 3 shows the adjusted HR (95% CI) of heat-related illnesses according to income levels. Overall, low-income patients were more likely to be susceptible to heat-related illnesses compared to those with middle and high incomes. After adjustment for age (stratified by 5-year intervals), sex, smoking status, alcohol consumption, exercise, BMI categories, high blood pressure, hyperglycemia, and local income per capita, the adjusted HR for heat-related illnesses were significantly higher for low-income groups (adjusted HR = 1.103; 95% CI: 1.022–1.191) than high-income groups. On the other hand, stratification analysis according to age (20–64 years and over 65 years) and sex (male and female) showed no difference in the likelihood of developing heat-related illnesses according to income levels.

**Fig. 4:**
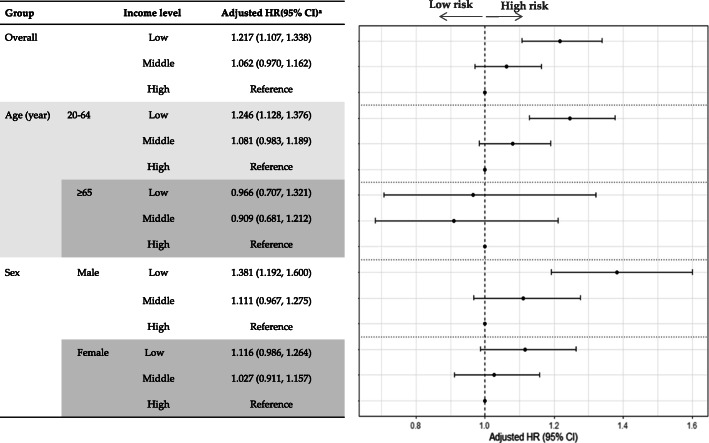
Adjusted hazards for cold-related illnesses according to income levels. ^a^ Adjusted model was controlled for age, sex, smoking status, alcohol drinking, exercise, BMI group, hypertension, hyperglycemia, and local income per capita

Figure 4 shows the adjusted HR (95% CI) of cold-related illness according to income levels. After adjusting for other covariates, participants with low income were likely to have 1.217 (95% CI: 1.107–1.338) times greater risk compared to those with high income. In the stratified analysis of age and sex, an HR for cold-related illnesses was higher in patient aged 20 to 64 years than in those aged over 65 years. Among those aged 20 to 64 years, an HR of 1.246 (95% CI: 1.128–1.376) was reported for low-income individuals. However, among those over 65 years, no significant increase in the risk for cold-related illness was noted. Male with low income had also a higher HR for cold-related illnesses than female with low income. An HR was only significant for low-income males (adjusted HR = 1.381; 95% CI: 1.192–1.600).

## Discussion

The health risks of extreme heat or cold are increasingly becoming evident [11]. Our concern was to explore whether individual income could affect differential risks of heat- and cold-related illnesses. Using a large, nationwide cohort of South Korea, we analyzed the associations between income and heat- or cold-related illnesses. This study indicates that people with low income were more susceptible to heat/cold-related illnesses than those with high income. Specifically, people with the lowest income had significantly increased hazard for heat- (adjusted HR = 1.103; 95% CI: 1.022–1.191) and cold-related illness (adjusted HR = 1.217; 95% CI: 1.107–1.338) than those with the highest income. Based on the current results, we suggest that low-income populations may be at risk of illnesses from extreme heat and cold events.

Extreme temperature poses a clear health impact, including excess morbidity and mortality [8, 11–17, 21–24]. The impact was heterogeneous across socioeconomic levels, which partly differed by unequal income [8, 10, 17, 25, 26]. Earlier studies, but not all [27, 28], had demonstrated that poverty- and income-related variables had a modifying effect on heat-mortality relationships [9, 11, 29–32]. Indeed, evidence has shown that dividuals with air conditioning had significantly reduced heat-related mortality [9, 33, 34]. In a recent analysis of the vulnerability to heat-related mortality, individuals with low SES exhibited significant greater risk relative to those with high SES [35]. Little research provides evidence on the effect of income on adverse cold-related health outcomes [28, 36, 37]. In the US, the age-adjusted cold-related death rates were about twice as much as in counties in the lowest median household income quartile than those in the highest median household income quartile from 2006 to 2010 [29]. Cold housing conditions and fuel poverty cause or exacerbate the risk of excess winter morbidity and mortality [36–38]. Taken together, extreme temperature-related health vulnerability among low income households remains inconclusive. However, considering evidence that access to air conditioning and sufficient fuel supply–usually a result of people having higher income–was protective against heat- and cold-related morbidity and mortality, it is unlikely to overlook unequal income in widening health disparities attributable to extreme temperatures.

Temperature is a critical variable for health and disease. The human body closely regulates core body temperature through a process called thermoregulation, keeping it steady at around 37 °C [18]. However, non-thermoneutral environments force thermoregulatory mechanisms compensate for extreme temperatures well above their thermal balance limits. For example, physiological response to heat stress is the cutaneous vasodilation, which liberates heat by radiant and convective heat loss, and sweating, which liberates heat by evaporation [18]. In response to cold stress, the autonomic nervous system coordinates cutaneous vasoconstriction, as well as metabolic and shivering thermogenesis, to retain bodily heat [18]. Under prolonged or extremely hot or cold external environments, the thermoregulatory capacity of humans becomes overwhelmed, making it difficult to maintain the body temperatures at normal levels. This places individuals at risk or life-threatening medical conditionss such as heat- and cold-related illnesses.

This study shows a health gap in heat- and cold-related illnesses according to individual income levels. Previous studies have suggested health vulnerability to extreme weather and climate change by individual- and community-level socioeconomic conditions (i.e., education, occupation, race, urban/rural, and green space) [8, 10–15, 17, 25, 26]. However, there is no study to suggest the effect of individual income on the occurrence of weather-related illnesses. Our study emphasizes the importance of focusing on “income levels” in developing extreme weather illnesses due to heat- or cold stress. The observed income-illnesses link is plausible and can be explained by various kind factors.

It is important to understand why low-income people are more frequently affected by heat- and cold-related illnesses than high-income people. While the exact mechanism for the observed association is beyond the current data, explanations on vulnerability to climate changes may be available to interpret our results. Vulnerability is the propensity or predisposition to be adversely affected by climatic risks depending on exposure, sensitivity, and adaptive capacity [6, 7]. Low income may present significant barriers to vulnerability reduction. Many studies suggest that the poor are less resistant to extreme weather events [4, 5, 7, 9, 11, 29–32]. This is because people with low income tend to live in housing with cheaper and less-desirable conditions (i.e., insulation and heating and cooling system), lack resources and options (i.e., private capital, food security, and time off), and have poor communication networks [39, 40]. Moreover, studies have shown that pre-existing medical conditions increase the risk of climate change-related diseases and death risks, which can be exacerbated by climate risks [41]. Notably, low-income individuals are more likely to suffer from chronic diseases [3]. Taken together, all these unequal distributions to resources, decision-making processes, and health conditions that low-income people face may put them at high exposure to extreme weather events, high sensitivity to health effects, and low adoptive capacity to handle climate risks, thus increasing their risk of climate-change related impact. Given these circumstances, our findings indicate that low-income people are particularly vulnerable to heat- and cold-related illnesses.

Several limitations should be considered. First, individual income encompasses the revenue streams from salaries, stock, rent, and interest on savings accounts. Given that income in the current study was solely based on average monthly household income, potential errors may exist in the interpretation of income disparities that lead to different health risks due to heat and cold exposure. Second, the definition of temperature-related illnesses was based on the ICD-10 codes, which could have underestimated the total burden of temperature-related mortality and morbidity caused by heat and cold exposure. Previous studies reported that analyses associated with heat-related and cold-related deaths based on the underlying causes derived from death certificates underestimated the number of deaths caused by exposure to heat or cold [29, 42, 43]. In the US national health report, including hyperthermia listed as contributing factors to the death certificate, heat-related deaths increased by 54%, and heat-related deaths were underestimated [42]. Weinberger et al. (2020) highlighted the lack of standardized criteria for identifying and recording death related to heat, as well as the difficulty of identifying cases where heat contributed to death from another cause (i.e., cardiovascular and respiratory diseases) [43]. Despite the differences in degree, defining heat- and cold-related illnesses based on ICD codes still creates problems related to diagnostic accuracy. However, it does serve as a reportable objective measure of disease burden from heat and cold. Third, patients with temperature-related illnesses were defined as those with the ICD-10 code diagnosed by healthcare facilities. However, in non-severe cases of illnesses, self-treatment at home could be made possible by purchase of treatment supplies without visiting a hospital. People with low income are less like to go to hospitals and thus record fewer temperature-related illnesses. This limits the ability to compare patients with like illness. We cannot rule out a potential misclassification or misinformation of patients with heat- and cold-related illnesses. Fourth, although the NHIS-NSC is constructed to ensure the representativeness of the entire Korean population, this study included only subjects who got physical examination; thus, there may be a systematic difference between individuals who participated in physical examination and those not in NHIS-NSC. This makes the study sample less representative of the population. As well, individuals who actively receive physical examinations are more likely to visit their physician and be diagnosed with weather-related illnesses or may have different levels of participation in state-provided health examinations depending on their income. It may be the results are unique to those samples and would not generalize to and across other situations. Finally, despite our considerable sample size, available data on factors affecting the occurrence of extreme weather illnesses have been lacking. For example, the effect of income on heat and cold susceptibility is moderated by many other factors, including housing quality, proportion of green space or vegetation, proximity to water, and electricity consumption. We cannot rule out the possibility of residual confounding variables. In view of these limitations, the results should be generalized with caution.

## Conclusions

We found that heat- or cold-related illnesses were more prevalent in Koreans with low income than those with high income. The differences in weather-related illnesses between the rich and poor were largely consistent with age (adults and elderly) and sex. This study suggests that individual income may be a risk factor for weather-related illnesses, and low income may exacerbate health disparities within the illnesses. In an era of climate change, understanding which factors create significant vulnerability to climate-related health is important to reduce health burdens of extreme temperatures. Our findings provide evidence of an association between income and weather-related illnesses, which could be useful in establishing appropriate strategies to reduce future health risks from exposure to extremely hot or cold temperatures. The present findings emphasize the importance of strategies relying on individual’s socioeconomic status, especially for lower-income groups. Ultimately, systematic measures such as, energy welfare programs to ensure proper cooling and heating, and evacuation facilities to alleviate damage from extremely hot or cold-related diseases, may help strengthen the adaptability of low-income households and reduce the high burden caused by heat or cold exposure.

## Data Availability

The data that support the findings of this study are available from the National Health Insurance Service in Korea but restrictions apply to the availability of these data, which were used under license for the current study, and so are not publicly available. Data are however available from the authors upon reasonable request and with permission of the National Health Insurance Service in Korea.

## Abbreviations

BMI: Body mass index
CI: Confidence interval
NHIS-NSC: National Health Insurance Service-National Sample Cohort
ORs: Odds ratios
SES: Socioeconomic status
